# Effects of Low Doses of Pioglitazone on Resting-State Functional Connectivity in Conscious Rat Brain

**DOI:** 10.1371/journal.pone.0117973

**Published:** 2015-02-11

**Authors:** Donna G. Crenshaw, Karen Asin, William K. Gottschalk, Zhifeng Liang, Nanyin Zhang, Allen D. Roses

**Affiliations:** 1 Zinfandel Pharmaceuticals, Inc., Chapel Hill, NC, United States of America; 2 Takeda Development Center Americas, Inc., Deerfield, IL, United States of America; 3 Biomedical Engineering Department, The Pennsylvania State University, University Park, PA, United States of America; University of Queensland, AUSTRALIA

## Abstract

Pioglitazone (PIO) is a peroxisome proliferator-activated receptor-γ (PPARγ) agonist in clinical use for treatment of type 2 diabetes (T2DM). Accumulating evidence suggests PPARγ agonists may be useful for treating or delaying the onset of Alzheimer’s disease (AD), possibly via actions on mitochondria, and that dose strengths lower than those clinically used for T2DM may be efficacious. Our major objective was to determine if low doses of pioglitazone, administered orally, impacted brain activity. We measured blood-oxygenation-level dependent (BOLD) low-frequency fluctuations in conscious rats to map changes in brain resting-state functional connectivity due to daily, oral dosing with low-dose PIO. The connectivity in two neural circuits exhibited significant changes compared with vehicle after two days of treatment with PIO at 0.08 mg/kg/day. After 7 days of treatment with a range of PIO dose-strengths, connections between 17 pairs of brain regions were significantly affected. Functional connectivity with the CA1 region of the hippocampus, a region that is involved in memory and is affected early in the progression of AD, was specifically investigated in a seed-based analysis. This approach revealed that the spatial pattern of CA1 connectivity was consistent among all dose groups at baseline, prior to treatment with PIO, and in the control group imaged on day 7. Compared to baseline and controls, increased connectivity to CA1 was observed regionally in the hypothalamus and ventral thalamus in all PIO-treated groups, but was least pronounced in the group treated with the highest dose of PIO. These data support our hypothesis that PIO modulates neuronal and/or cerebrovascular function at dose strengths significantly lower than those used to treat T2DM and therefore may be a useful therapy for neurodegenerative diseases including AD.

## Introduction

There is neither a cure nor an approved disease-modifying therapy for late-onset Alzheimer’s disease (AD). Given the global burden of AD and the anticipated increase in its incidence, there is intense interest in developing therapies that will prevent the onset of the disease or delay its progression. According to one model, if a therapy that delayed onset of AD by 5 years were introduced in 2015, the prevalence of AD in the United States would be reduced by 50% in 2050 [1]. Therapy would be particularly beneficial if it were initiated before the onset of any cognitive symptoms and at earlier ages for those at risk of developing late-onset AD.

Pioglitazone (PIO) is an insulin-sensitizing agent marketed as ACTOS for the treatment of T2DM. For this indication, PIO is used daily at strengths of 15, 30, or 45 mg. PIO is a member of the thiazolidinedione class of PPARγ agonists [2]. PPARs are ligand-activated transcription factors; the γ-isotype is expressed in a number of human tissues including muscle, fat, liver, and brain [3]. The activated transcription factor binds as an obligatory heterodimer with the retinoid X receptor-α (RXRα) and regulates transcription of many genes, including genes involved in adipogenesis, insulin sensitivity, and inflammation [2]; it also regulates mitochondrial biogenesis in adipocytes [4], in human neuroblastoma cells [5], and in the brains of mice [6].

In addition to being effective antidiabetic agents [7], PPARγ agonists may be useful for treating neurodegenerative diseases, including AD [8]. Two commercially available agonists, rosiglitazone (ROSI) and PIO, have been tested in transgenic mouse models of autosomal dominant, early-onset AD and have produced a number of salutary effects, including reducing soluble brain Aβ or Aβ plaques, limiting microglial activation and the expression of inflammatory proteins or transcripts in the brain, improving neurometabolic and neurovascular coupling responses, and improving cognition (reviewed in [9,10]).

A small number of open-label and double-blinded, randomized, human clinical trials have tested PPARγ agonists for the treatment of AD dementia or mild cognitive impairment with varying degrees of success [11–17]. For example, in addition to demonstrating a reduction in the rate of cognitive decline, one study showed that adding 15–30 mg/day PIO to the treatment regimen of T2DM patients (n = 21) with mild to moderate AD dementia or amnestic MCI improved cerebrovascular function relative to a T2DM-treatment regimen without PIO [16]. Results from *in vitro* studies also suggest that lower doses of ROSI or PIO than those used to treat T2DM may be more efficacious for AD treatment, including protecting against the development of the disease [5,18–20]. In addition, since these cited clinical trials were completed, it has been proposed that treatment of AD, including treatment with PPARγ agonists, may be more successful if initiated earlier in the disease process when AD pathology is less developed [12,21].

Functional magnetic resonance imaging (fMRI) measures the blood-oxygenation-level dependent (BOLD) signal that is associated with neural activity [22]. When the brain is resting, low-frequency spontaneous fluctuations of the BOLD signal in specific brain regions are temporally correlated and organized into functional networks [23,24]. Perturbations in resting-state functional connectivity (RSFC) have been associated with disease states including AD [25,26], and this technique has also been used to track the effects of drugs on brain function [27]. Resting-state fMRI experiments have been conducted in non-anaesthetized rats and have shown that rats possess functional networks that are topologically similar to those in human brains [28–31]. The rat, therefore, provides a viable model in which to test the effects of drugs on brain function.

In preclinical species, the blood brain barrier is reported to be relatively impermeable to PIO at oral doses of 0.5 mg/kg [32]. Earlier studies of PIO on mouse models of autosomal dominant AD have used high doses of the drug (see [33] for example); the effects of low doses of PIO on brain activity have not been examined. Therefore, we asked whether low doses of this drug affected brain activity, in non-diabetic rats using RSFC. This technique has many advantages, and specifically for our purposes is that it depends upon and is a surrogate measure for brain hemodynamics and energetics (glucose extraction and metabolism) which are perturbed during the development of AD [34,35]. Our results provide preliminary evidence that PIO, at doses much lower than those used to treat T2DM, can elicit changes in RSFC. Moreover, these preliminary results suggest that the drug, at doses in the range of that being used in an on-going Phase III clinical study examining efficacy of PIO in delaying the onset of AD, impact connections between areas of the brain that are affected early in the development of AD.

## Materials and Methods

### Animal husbandry and drug preparation

Adult male Wistar rats (300 ± 50 g upon arrival) from Charles River (Wilmington, MA) were housed separately in Plexiglas cages and maintained at 22–24 °C on a 12-h light/dark schedule. Food and water were provided *ad libitum*. All animals were acquired and cared for in accordance with the guidelines published in the *Guide for the Care and Use of Laboratory Animals (National Institutes of Health Publications No*. *85–23*, Revised 1985). All efforts were made to minimize animal suffering, including light anaesthesia with isoflurane to facilitate drug administration and to secure the animals in the restraints and using topical anesthetic to reduce possible discomfort from the restraints. The protocol was approved by the IACUC Committee of the University of Massachusetts Medical School (IACUC protocol number 2401–12).

PIO (Takeda Development Center Americas, Inc.) was dissolved in 0.5 mol/L citric acid to yield a stock solution of 0.32 mg PIO/10 mL. Other dosages were prepared by dilution of the stock solution with 0.5 mol/L citric acid to yield dose volumes of 10 mL/kg. Each dose group consisted of 5 or 6 rats. Control rats received the vehicle at 10 mL/kg. Body weights were determined every 5 days throughout the course of the experiment, and the amount of PIO solution administered was adjusted according to the most recent body weight to achieve doses of 0.04, 0.08, 0.16, or 0.32 mg/kg/day. Drug administration was by oral gavage at approximately the same time every day. Animals were anesthetized lightly with isoflurane to facilitate drug administration.

There were two arms to this study, the Acute and Sub-chronic, distinguished primarily by the number of days of drug treatment prior to the final fMRI scan that was analyzed (Table 1). All rats were scanned on Study Day-3 (SD-3) following administration of vehicle (0.5 mol/L citric acid). This constituted the ‘baseline’ scan. The first day of treatment was three days later, on Study Day 1 (SD1). In the Acute arm of the study, one group of vehicle- and one group of PIO-treated (0.08 mg/kg/day) rats were scanned after the second dose of PIO on SD2. Although these rats were also scanned on SD7, the data from this scan of these animals were not analyzed. While all rats (from the Acute Arm and Sub-chronic Arms) were scanned after the last daily dose of the vehicle or PIO on SD7, the scans from only the rats in the Sub-chronic arm of the study were analyzed for the SD7 time point. This was done to eliminate any effect due to an intermediate MRI scan. Excluding the baseline scan, all scans were initiated approximately 2.5–3 hours after dosing of placebo or PIO. This elapsed time coincided approximately with the time of peak plasma concentrations (Tmax) of PIO in rats [32].

**Table 1:** Treatment arms, daily PIO dose, and imaging time points.

Treatment arm	Daily dose (mg PIO/kg)	Imaging time points		
		SD-3	SD2	SD7
**Acute**	0*, 0.08^†^	✓	✓	✓
**Sub-chronic**	0*, 0.04^†^, 0.08^†^, 0.16^†^, 0.32*	✓	No imaging	✓

*n = 6/group;

^†^n = 5/group (Total n = 38)

### Animal restraint and acclimation

Under short-acting anesthesia (2.5% isoflurane for 3–5 minutes), a topical anesthetic (EMLA cream, AstraZeneca) was applied to the ear canal and bridge of the nose. A plastic semicircular headpiece was positioned using blunted supports inserted in the ear canals. The head was placed into the cylindrical head-holder with the canine teeth of the rat secured over a bite bar, the nose was secured with a nose clamp, and the ears positioned inside the head-holder with adjustable screws fitted into lateral sleeves. The animal’s forepaws and hind paws were loosely taped to prevent self-injurious behavior. The administration of isoflurane was stopped as soon as the animal was secured. An adjustable surface coil built into the head holder was pressed firmly onto the head and locked into place. The body was restrained in a sleeve that was suspended down the center of the chassis and buffered by rubber gaskets. The head holder was locked to a mounting post. A volume coil was locked into position over the head holder. This procedure took 10–15 minutes, at the end of which time the animal was usually fully conscious. The restrained rat was then placed into a Plexiglas body tube and the entire unit was secured onto a firm base to prevent motion, and this was then placed in the MRI scanner.

Prior to the start of the imaging phase of the protocol, rats were gradually conditioned to the scanner environment, including the scanner noise. Animals restrained as described earlier were placed in a black opaque tube (mock scanner) for 15 minutes on the first day, increasing by 15 minutes per day to a maximum time of 90 minutes on days 6 and 7 of the acclimation period. A recording of sounds from various imaging sessions was played throughout the acclimation phase. Previously, we (ZL, NZ) showed that these restraint and acclimatization procedures minimized animal stress during MRI scanning [36,37], and we have successfully used them in a number of other studies [28–31,38].

After acclimation, animals were assigned to 1 of 7 treatment arms matched for mean body weights.

### Magnetic resonance imaging

Functional MRI was conducted using a 4.7T/40cm horizontal magnet (Oxford, UK) interfaced with a Biospec Bruker console (Bruker, Germany) and equipped with a 20G/cm magnetic field gradient. A dual 1H radiofrequency (RF) coil configuration (Insight NeuroImaging Systems, Worcester, MA) was used; it consisted of a volume coil for exciting the water proton spins and a surface coil (2 cm) for receiving MRI signal. The volume and surface coils were actively tuned and detuned to prevent mutual coil coupling. This dual-coil configuration allows for sufficient homogeneity in the radio frequency transmission field in the rat brain, while preserving the advantage of a higher signal-to-noise ratio provided by the smaller reception coil.

We conducted imaging only on conscious animals. Initially, anatomical images of the head of each restrained animal were acquired using a multi-slice fast spin-echo sequence (RARE) with the following parameters: repetition time (TR) = 2125 ms; RARE factor = 8; effective echo time (TE) = 50 ms; matrix size = 256 × 256; field of view (FOV) = 3.2 × 3.2 cm^2^; slice number = 18; slice thickness = 1 mm; n = 8. Based on the geometry of the anatomical images, multi-slice gradient-echo images covering the whole brain were acquired using echo-planar imaging (EPI) with the parameters: TR = 1 s; Flip Angle = 60°; TE = 30 ms; matrix size = 64 × 64; FOV = 3.2 × 3.2 cm^2^; slice number = 18; slice thickness = 1 mm. Two hundred volumes were acquired for each run; 9 runs were obtained for each scan session.

### Data analysis and preprocessing of imaging data

Body weight data across the treatment period were analyzed using a 2-way (Day x Treatment) repeated measures analysis of variance (ANOVA). For some analyses of body weight, data from the Acute and Sub-chronic groups were pooled.

The fMRI data were pre-processed using Medical Image Visualization and Analysis (MIVA http://ccni.wpi.edu), Statistical Parametric Mapping (SPM8) software (Wellcome Department of Cognitive Neurology, London, UK), and Matlab (The Mathworks Inc., Natick, MA, USA). The data were initially corrected for motion (threshold of 0.5 mm). The average of the group means for motion (and average of the mean SEM across groups) was 0.053±0.008 mm. The average of the means across all groups for proportion of sessions rejected was 21.1%. Further pre-processing of data included spatial smoothing using a 3D Gaussian filter (1 mm full width at half maximum) and band-pass (0.0017–0.1Hz) filtering. Structural and functional data for each animal were transformed to a standard stereotaxic space embedded in MIVA to facilitate group analysis of functional data [28,29,31].

We performed correlational functional connectivity analysis to evaluate RSFC between pairs of regions of interest (ROI). Each animal was aligned and co-registered, based on anatomical images, to a fully segmented rat brain atlas [39]. The co-registration procedure provided the coordinates of each ROI in the image space. After co-registration and alignment of anatomical images from all rats, fMRI time courses for individual voxels in a seed ROI were obtained according to their corresponding coordinates. A time course for each seed region was then obtained by regionally averaging time courses from all voxels inside the seed ROI.

**Fig 1:**
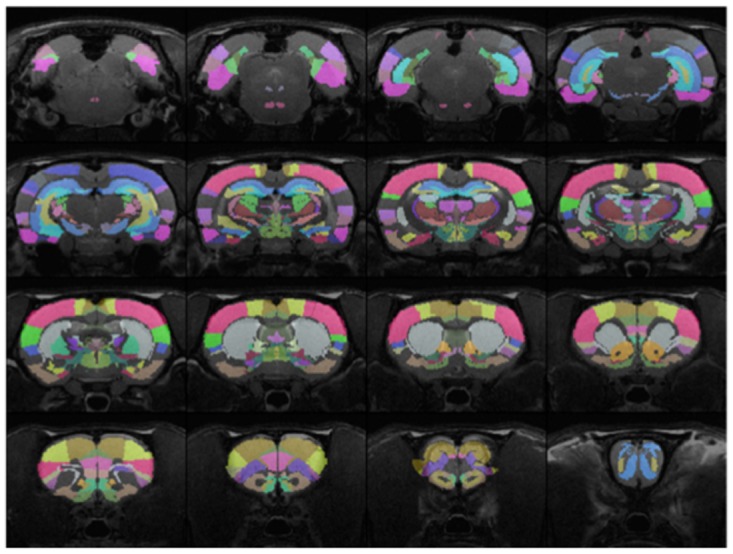
The 57 ROIs used in the assessment of whole brain functional connectivity. The regions were identified according to a fully segmented rat brain atlas [39].

**Network-based analysis**. To evaluate the effects of PIO on functional connectivity across the whole brain, the rat brain was divided into 57 ROIs (Fig. 1). We evaluated the strength of functional connectivity between each pair of ROIs (*i*.*e*., each connection) by determining the cross-correlation coefficient between two ROI fMRI time courses. A total of 57 × 56/2 = 1,596 functional connections were assessed during each rsfMRI run. The procedure was repeated for 9 runs during each MRI session for every animal. The connectivity strength reported for each connection was the average of the cross-correlation coefficients for the 9 runs. A 2-way (Day × Treatment) ANOVA with repeated measures on the Day factor was conducted for each connection. The statistical significance level was set at *p* < 0.005, uncorrected. The treatment groups were analyzed separately to evaluate the effect of 2 (Acute) and 7 (Sub-chronic) daily treatments with PIO.

**Seed-based analysis**. Seed-based correlational analysis was also conducted to evaluate the effects of PIO on neural circuits of a designated ROI (the CA1 region of the hippocampus). The spatial patterns of brain regions that were functionally connected with the seed ROI were calculated in a voxel-by-voxel manner. First, the regionally-averaged time course of the seed ROI was obtained as a reference. The cross-correlation coefficient between the time course of each voxel and the reference time course was then calculated. The cross-correlation coefficient represented the functional connectivity strength between voxel and the seed. A connectivity map for the seed ROI was created for each fMRI run and maps from nine runs were then averaged to create the connectivity map for each scan session. Finally, a composite connectivity map was generated by averaging connectivity maps across rats of the same dose group that were imaged on the same dose day. More details of seed-based correlational analysis are described elsewhere [29–31]. Since the voxel-by-voxel type of analysis requires much higher statistical power due to the large number of comparisons (total comparisons = 64 × 64 × 18 = 73,728) and the small sample size in in each dose group, this study is under-powered to obtain statistical significance after correcting for multiple comparisons. Therefore, the seed ROI results are qualitative. The seed-based analysis was conducted only for the Sub-chronic groups (*i*.*e*. those treated for 7 days).

## Results

### Body weights

All rats were weighed at baseline (SD-3), SD2, and SD7. Mean group weights are reported in Table 2. A two-way, repeated measures ANOVA (Day × Treatment) conducted on the body weights revealed that there was a significant effect of the Day factor in the Sub-chronic groups (*p* = 0.008) and the pooled Sub-chronic and Acute groups (*p* = 0.002), but not in the Acute groups (*p* = 0.09). While there was a significant effect of the Day factor on body weight, PIO treatment was not a significant factor in change in body weight over Days (Sub-chronic, *p* = 0.91; Acute, *p* = 0.64; and pooled groups, *p* = 0.66).

**Table 2:** Mean (SD) body weights for each treatment group at baseline and on each imaging day.

	Body weight (g) (±SD)		
Treatment arm (Dose, mg PIO/kg)	SD-3	SD2	SD7
Acute (0)	321.4 (5.2)	324.4 (5.9)	324.6 (6.7)
Acute (0.08)	327.3 (10.3)	331.0 (11.7)	332.3 (13.1)
Sub-chronic (0)	333.7 (6.5)	348.5 (8.5)	341.7 (9.1)
Sub-chronic (0.04)	345.6 (8.6)	353.0 (12.2)	352.8 (14.8)
Sub-chronic (0.08)	331.8 (10.0)	338.8 (14.7)	335.2 (17.6)
Sub-chronic (0.16)	334.6 (11.7)	342.4 (16.2)	343.4 (16.4)
Sub-chronic (0.32)	340.2 (6.9)	346.7 (10.6)	348.3 (11.4)

### Effects of PIO on whole brain functional connectivity

Whole brain functional connectivity was assessed after 2 or 7 treatments. After 2 treatments with 0.08 mg PIO/kg/day, two functional connections, between the tegmental reticular nucleus in the pontine gray and the prelimbic area, and between the CA3 field of the hippocampus and the lateral septal complex, were significantly changed (*p* < 0.005, uncorrected) from baseline and relative to the vehicle control (Table 3).

At SD7, an ANOVA conducted on the cross-correlation coefficients between ROIs indicated that 17 connections were significantly different (*p* ≤ 0.005, uncorrected) at SD7 relative to baseline across the 5 Sub-chronic dose groups. These significant changes in functional connectivity were between the brain regions indicated in Table 4. However, none of the changes was significant after using the correction for multiple comparisons at a false discovery rate adjusted *p*-value of 0.05, likely due to the small sample size and limited statistical power.

**Table 3:** Change in functional connectivity following two doses of 0.08 mg PIO /kg in the Acute arm of the study.

Region 1	Region 2	F_interaction_ (df = 1,9)	*p*_interaction_
CA3 field of the hippocampus (CA3)	lateral septal complex (LSX)	28.69	0.0010
tegmental reticular nucleus, pontine gray (TRN)	prelimbic area (PL)	18.02	0.0038

The ANOVA revealed a significant (*p* ≤ 0.005) Day × Treatment interaction for functional connections between two pairs of ROI even at this early time point.

**Table 4:** Change in functional connectivity after 7 days of treatment.

Region 1	Region 2	F_interaction_ (df = 4,22)	*p*_interaction_
midline group, dorsal thalamus (MTN)	primary somatosensory area (SSp)	9.2705	0.0002
prelimbic area (PL)	primary somatosensory area (SSp)	7.0645	0.0008
lateral nuclei, dorsal thalamus (LAT)	globus pallidus (GP)	7.0647	0.0008
secondary somatomotor areas (MOs)	supplemental somatosensory area (SSs)	6.8393	0.001
secondary somatomotor areas (MOs)	primary somatosensory area (SSp)	6.537	0.0013
lateral nuclei, dorsal thalamus (LAT)	reticular nucleus thalamus (RT)	6.2181	0.0017
prelimbic area (PL)	supplemental somatosensory area (SSs)	6.0162	0.002
primary somatomotor area (MOp)	orbital area (ORB)	5.8993	0.0022
tegmental reticular nucleus, pontine gray (TRN)	primary somatomotor area (MOp)	5.6476	0.0028
supplemental somatosensory area (SSs)	ectorhinal area (ECT)	5.651	0.0028
orbital area (ORB)	primary somatosensory area (SSp)	5.5135	0.0031
amygdala (AMG)	substantia nigra (SN)	5.4787	0.0032
anterior cingulate area (ACA)	supplemental somatosensory area (SSs)	5.5055	0.0032
lateral nuclei, dorsal thalamus (LAT)	anterior cingulate area (ACA)	5.4452	0.0033
gustatory area (GU)	auditory areas (AUD)	5.3472	0.0037
tegmental reticular nucleus, pontine gray (TRN)	secondary somatomotor areas (MOs)	5.1488	0.0044
primary somatomotor area (MOp)	supplemental somatosensory area (SSs)	5.0451	0.0049

The ANOVA revealed a significant (*p* ≤ 0.005) Day x Treatment interaction for functional connections between 17 pairs of ROI across the rat brain.

### Effects of PIO on hippocampal functional connectivity

**Fig 2:**
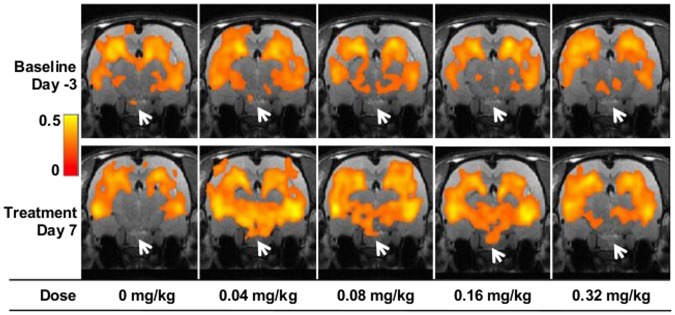
Functional connectivity map from the hippocampus seed for each PIO dose group from the Sub-chronic arm of the study on SD7. Functional connectivity strength is presented as a heat map of the cross-correlation coefficient. PIO treatment most noticeably influences the functional connections between regions broadly indicated by the white arrows, including the hypothalamus and ventral thalamus, and the hippocampal CA1 region.

In a *post hoc*, seed-based analysis, single neural circuits were investigated using the CA1 region of the hippocampus as the seed ROI for rats in the Sub-chronic arm of the study [31]. Maps of brain areas that were functionally connected to the CA1 region at baseline and day 7 of treatment are shown in Fig. 2 for all Sub-chronic dose groups and the Control group. The overall pattern of CA1 connectivity was consistent among all groups at baseline and in the Control group on SD7. Compared with baseline, the most obvious and consistent change in the PIO-treated groups on SD7 was the increased connectivity between the CA1 region and the hypothalamus and the ventral thalamus (Fig. 2). The increased connectivity was greatest following treatment with PIO at 0.04, 0.08, 0.16 mg/kg/day, but was relatively less following treatment with the highest PIO dose, 0.32 mg/kg/day.

## Discussion

PPARγ agonists have been proposed for the treatment of late-onset AD and a small number of open-label and double-blinded, randomized trials have been conducted with ROSI or PIO in patients afflicted with mild to moderate disease. These trials have had mixed success, although hints of beneficial effects were evident even in failed studies [11–17].

With advances in our understanding of AD, it has become apparent that the disease process begins years, or even decades, before the first clinical signs of AD appear [40]. This improved understanding has led the AD community to posit that a therapeutic intervention may have more potential for success if treatment is initiated earlier in the disease course, preferably during the pre-symptomatic phase. In light of emerging preclinical and clinical data and new hypotheses, Takeda Development Center Americas and Zinfandel Pharmaceuticals are collaborating to assess the efficacy of low-dose PIO in delaying the onset of mild cognitive impairment (MCI) due to AD in individuals who are cognitively normal at the initiation of treatment (ClinTrials.gov identifier NCT01931566). The treatment population for the Phase 3 clinical trial will be enriched with subjects at high risk for developing AD within the next several years based on a risk algorithm comprised of genetic factors, *APOE* and *TOMM40* genotypes, and subject age [41]. The dose of PIO being evaluated in the clinical trial is much lower than the lowest dose approved for the treatment of T2DM.

In this study, we used RSFC, across the whole brain or with the CA1 region of the hippocampus as a seed region, as a sensitive measure of brain function before and after treatment with PIO. The whole-brain rsfMRI analysis suggests that low-dose PIO elicits changes in rat brain functional connectivity at least at Tmax [32] following 2 and 7 days of treatment. On SD2, two functional connections were different from baseline and from Control (Table 3). An ANOVA revealed that, following 7 days of treatment, 17 functional connections were significantly changed from baseline in at least one treatment group (Table 4).

In the seed ROI analysis, 7 days of PIO treatment with daily doses ranging from 0.016 mg/kg to 0.32 mg/kg qualitatively increased the correlation between the seed ROI, the CA1 region of the hippocampus, and the ventral thalamus and hypothalamus relative to the baseline for each group and relative to the Control group. Apart from treatment with vehicle alone, the smallest change in functional connectivity with the CA1 region occurred at the highest PIO dose tested, 0.32 mg/kg/day. Although this trend bears replication, it does suggest that the relationship between PIO dose and functional connectivity with the CA1 region may be described by an inverted-U shaped function. This result is consistent with a recent report by Moon *et al*., who demonstrated that expression of the LDL receptor related protein 1 (LRP1), which mediates transcytosis of Aβ across the blood brain barrier [42], is maximally induced in cultured human brain microvascular endothelial cells at low concentrations of ROSI, and expression decreases to baseline levels at higher drug concentrations [19]. A similar effect was seen with PIO; maximal LRP1 protein expression occurred at 50 nM PIO and decreased to approximately baseline levels at 100 nM PIO [19]. Miglio *et al*. earlier showed that low concentrations of PIO and ROSI (maximal effect at ~100 nM) induced expression of mitochondrial transcription factors (NRF1 and TFAM), the transcription co-activator PGC-1α, PPARγ itself, electron transport chain components (cytochrome c oxidase subunits I and IV), and mitochondrial DNA content in cultured neuroblastoma cells, and protected the cells against glucose deprivation-induced cell death [5]. Although the effects were not diminished at higher drug concentrations, they did plateau. Nanomolar concentrations of PIO also stimulated neurite outgrowth in neuroblastoma cells and maximal effects also were observed at ~100 nM [18]. These results suggest that low PIO concentrations positively impact a number of molecular pathways that become impaired or dysregulated in neuronal cells during the development of AD, leading to, for example, changes in mitochondrial biogenesis, Aβ accumulation, and neurite degeneration [19,43].

Whether the observed changes in the neural circuitry in this study are due to direct effects of PIO on the brain or are downstream effects of drug modulation of peripheral targets is not known. However, the drug target, PPARγ, is expressed in neuronal and non-neuronal cell types, including microglia, in multiple regions of the brain including the CA1, CA3 regions and dentate gyrus of the hippocampal formation, the medial thalamus, and the hypothalamus [44–47]. RXR, the obligate binding partner for PPARγ, is also widely expressed in brain, including regions that express PPARγ [48,49]. In addition, it has been directly shown that PIO crosses the blood-brain barrier, albeit quite poorly [32]. Therefore, the most parsimonious explanation is that the changes in RSFC we observed are due to direct effects of PIO-activated PPARγ on target genes in the brain.

Although it is formally possible that the changes in functional connectivity seen in this experiment are due to stress invoked by the procedure, we think that this is unlikely. Animal stress during the scanning procedure is minimized by using an entirely noninvasive restraining system and a routine acclimation procedure. Previous studies have shown that the acclimation and imaging procedures are not likely to induce chronic stress in animals. For example, King *et al*. reported that various physiologic variables of rats were reduced following 5–8 days of acclimation [36], which is the same acclimation regimen adopted in the current study. In their study, most measures of stress including corticosterone levels were reduced near to pre-stress baseline levels on day 4–5 of the acclimation regimen. Similarly, Liang *et al*. showed that the procedure used in this study to acclimate the rats to the MRI restraint and noise did not result in significant stress as measured by behavior in an elevated plus maze test [37].

To control for motion during imaging, the threshold for displacement between any two consecutive rsfMRI volumes across any rsfMRI run was set at 0.5mm. This was the largest observed displacement and this threshold has been used in previous studies [28–30,37,38,50]. The effect of motion on RSFC was recently investigated by some of the authors on this study (NZ, ZL) who found that decreasing the motion threshold to a level seen with anesthetized rats did not significantly change the observed RSFC patterns [29,38]. Taken together, we believe the motion threshold applied in the present study is justified.

Other potential artifacts were controlled for during image pre-processing. In particular, we filtered out high-frequency signals by setting the threshold of the low-pass filter at 0.1 Hz. As a result, any contribution to the observed RSFC from respiratory and cardiac processes should be greatly diminished. To further minimize the any influence of physiologic noise, during image preprocessing we also regressed out the signals from the white matter and ventricles that are presumably dominated by physiologic noise [51]. In addition, a recent publication demonstrated that in the rat the relative contributions from cardiac and respiratory noise to the rsfMRI signal were only 1% and 5%, respectively [52]. Therefore, with the stringent data analysis procedures that were applied in order to control for potentially small physiologic effects, and because all animals including the control groups were treated in the same way, we believe that the impact of physiologic noise on our results should be minimal.

Resting state-fMRI is gaining recognition as an important tool for AD research, and may be particularly useful for evaluating changes that occur in preclinical AD and in the progression to early clinical stages of the disease [53,54]. For example, this technology has provided evidence that functional connections in relatively young individuals and in cognitively normal elderly adults who carry the *APOE* ε4 risk allele for AD are distinct from those of individuals who do not carry the risk allele [55], and that differences are seen in advance of the deposition of amyloid or altered Aβ42 in the cerebrospinal fluid [56]. Functional connectivity within the default mode network, a network that is active when the brain is not challenged with a task and includes connections to and within the hippocampus, becomes aberrant in MCI and in AD [54]. Although speculative, the observed trend for enhancement in functional connectivity with the CA1 that is seen in this rat study is particularly interesting in light of earlier findings on disease-related changes in functional connectivity, and because whole-brain functional networks in rats are organized into topologies that are similar to those in the human brain [28].

When used for T2DM, PIO treatment results in mild weight gain by inducing fluid retention and/or by expanding subcutaneous fat. These effects are positively correlated with drug dose [57] but may not occur at doses lower than those marketed for treatment of T2DM [58]. In the present experiment, low doses of PIO did not significantly impact body weight in the rats since average group weights increased by a similar amount in all groups, including the two control groups (Table 2). In addition, most of the weight gain that was observed, including that of the Control group, occurred between SD-3 and SD2, after only 2 doses of drug. Whether drug-related weight gain would be expected after only a week of treatment is not known, but the result suggests that use of a low dose may reduce the likelihood of this adverse effect associated with this class of drugs.

The major limitation of this study is that it is underpowered; the sample size of each dose-group is small considering the large number of comparisons that are conducted. Small sample size is most problematic for the seed-based analysis, which is a *post-hoc* analysis of the available data. Nonetheless, our study successfully demonstrates the utility of using rsfMRI in conscious rats to test the pharmacodynamic effects of drugs on brain function in the absence of the potentially confounding effects of anesthesia. The results also support our hypothesis that low doses of PIO enter the brain and influence neural activity and do so in regions that are pertinent to the early pathogenesis of AD.
